# RNA interference-mediated knockdown of the hydroxyacid-oxoacid transhydrogenase gene decreases thiamethoxam resistance in adults of the whitefly *Bemisia tabaci*

**DOI:** 10.1038/srep41201

**Published:** 2017-01-24

**Authors:** Xin Yang, Wen Xie, Ru-mei Li, Xiao-mao Zhou, Shao-li Wang, Qing-jun Wu, Ni-na Yang, Ji-xing Xia, Ze-zong Yang, Li-tao Guo, Ya-ting Liu, You-jun Zhang

**Affiliations:** 1 Department of Plant Protection, Institute of Vegetables and Flowers, Chinese Academy of Agricultural Sciences, Beijing, 100081, P. R. China

## Abstract

*Bemisia tabaci* has developed a high level of resistance to thiamethoxam, a second generation neonicotinoid insecticide that has been widely used to control this pest. In this study, we investigated whether hydroxyacid-oxoacid transhydrogenase (*HOT*) is involved in resistance to the neonicotinoid insecticide thiamethoxam in the whitefly. We cloned the full-length gene that encodes *HOT* in *B. tabaci*. Its cDNA contains a 1428-bp open reading frame encoding 475 amino acid residues. Then we evaluated the mRNA expression level of *HOT* in different developmental stages, and found *HOT* expression was significantly greater in thiamethoxam resistance adults than in thiamethoxam susceptible adults. Subsequently, seven field populations of *B. tabaci* adults were sampled, the expression of mRNA level of *HOT* significant positive correlated with thiamethoxam resistance level. At last, we used a modified gene silencing system to knock-down *HOT* expression in *B. tabaci* adults. The results showed that the *HOT* mRNA levels decreased by 57% and thiamethoxam resistance decreased significantly after 2 days of feeding on a diet containing *HOT* dsRNA. The results indicated that down-regulation of *HOT* expression decreases thiamethoxam resistance in *B. tabaci* adults.

Neonicotinoid insecticides are active against numerous sucking and biting pest insects, including aphids, beetles, some lepidopteran species, and whiteflies^1^. The whitefly *Bemisia tabaci (B. tabaci*), a destructive pest of many protected crops and field crops worldwide, directly damages plants by feeding and indirectly damages plants by vectoring more than 100 plant viruses^2^. Control of *B. tabaci* in crop systems worldwide has largely depended on insecticides and especially on neonicotinoid insecticides. Thiamethoxam, a second-generation neonicotinoid insecticide discovered and developed by the Novartis Crop Protection, has been used extensively for management of *B. tabaci* in horticultural and other cropping systems^3^. High levels of resistance to thiamethoxam, however, have been recently reported for *B. tabaci* populations in Israel, Spain, and China^4^,^5^,^6^,^7^.

The most significant mechanisms of resistance to neonicotinoid insecticides are increased metabolic detoxification and decreased target-site sensitivity. A target site mutation of the nicotinic acetylcholine receptors (nAChR) (Y151S) was identified as the cause of target-site insensitivity to imidacloprid, the primary commercial neonicotinoid pesticide, in *Nilaparvata lugens* in the laboratory^8^. In addition, the discovery of a single mutation (R81T) in *Myzus persicae* and its association with the reduced affinity of the nAChR for imidacloprid is the first example of field-evolved target-site resistance to neonicotinoid insecticides^9^. With respect to metabolic detoxification, the cytochrome P450s play dominant roles in the metabolism of a wide variety of both endogenous and xenobiotic substances. Over-expression of a cytochrome P450, *CYP6CM1*, has been linked to imidacloprid resistance in *B. tabaci*^10^. Moreover, *CYP6CM1* and another P450 gene, *CYP4C64*, have been associated with imidacloprid resistance in field populations of *B. tabaci* in China^11^.

“Omics” analyses and a microarray have been used to examine differences between a thiamethoxam-resistant strain (THR) and a -susceptible strain (THS) of *B. tabaci* at both transcriptional and translational levels. The results showed that the expression of a suite of phase I and phase II detoxification enzymes, including cytochrome P450s, UDP-glucuronosyltrans-ferases, glutathione S-transferases (GST), and several ABC transporters was substantially elevated in THR^12^. More recently, the mechanism of thiamethoxam resistance in *B. tabaci* was investigated using the suppression subtractive hybridization (SSH) cDNA library approach. Based on the results of the differential screening, 298 and 209 clones were picked and sequenced, respectively, from the forward and reverse cDNA libraries, representing genes that were up- and down-regulated in THR relative to THS. Among the genes, one encoding a hydroxyacid-oxoacid transhydrogenase (*HOT*) EST (previously named as NAD-dependent methanol dehydrogenase-like EST) was substantially over-expressed (~12-fold) in the THR strain^13^. *HOT* catalyzes the cofactor-independent conversion of γ-hydroxybutyrate (GHB) to succinic semialdehyde (SSA) with coupled converting it to conversion of 2-oxoglutarate to D-2-hydroxyglutaric acid. Unlike many other alcohols, which are oxidized by NAD-linked dehydrogenases, GHB is metabolized to SSA by *HOT* without requiring free NAD and NADP^14^,^15^.

In the current study, we cloned the full-length *HOT* gene in *B. tabaci*, evaluated its mRNA expression level in different developmental stages, determined the resistance level of thiamethoxam in seven field population, and evaluated the relationship between resistance rate and the mRNA level expression of *HOT*. Furthermore, we used a modified gene silencing system to knockdown the *HOT* gene. We report that down-regulation of *HOT* decreases thiamethoxam resistance in *B. tabaci* adults.

## Results

### Gene cloning and analysis

**Figure 1:**
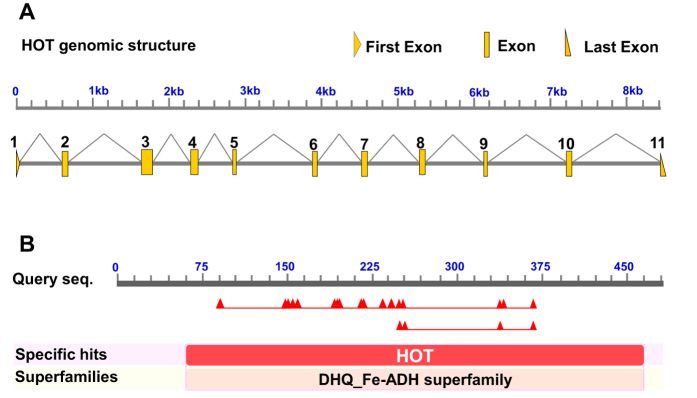
Genomic structure of *HOT* and blast result in NCBI database. (**A**) Genomic sequence revealed that *HOT* contains of 11 exons that encode 475 amino acids protein. (**B**) The deduced amino acid sequence contains important conserved domains that are common to other *HOT* members.

**Figure 2:**
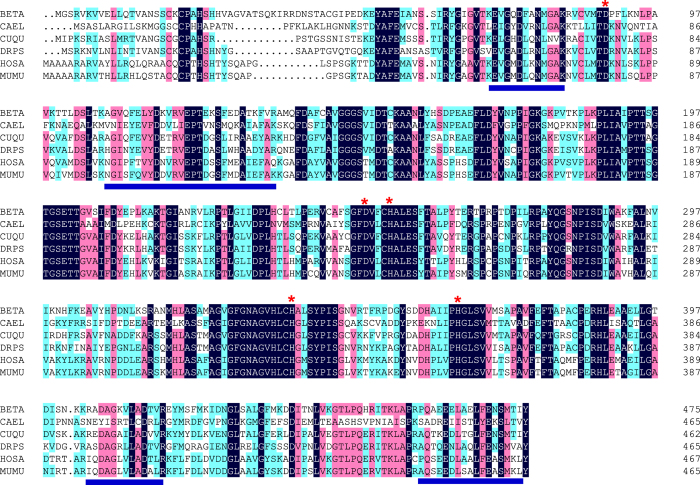
Putative *HOT* sequences from *Caenorhabditis elegans* (Cael; GI75025507), *Culex quinquefasciatus* (Cuqu; GI167882237), Drosophila *pseudoobscura* (Drps; GI54636830), *Homo sapiens* (Hosa; GI25989126), and *Mus musculus* (Mumu, GI37589962). The conserved residues are indicated by a black background. Asterisks indicate the position of Asp89 (which discriminates against NADP binding) and of Asp250, His254, His335, and His365 (metal-binding residues). The blue underlined residues in the mouse sequence correspond to the peptides identified by mass spectrometry in the enzyme purified from rat liver.

**Figure 3:**
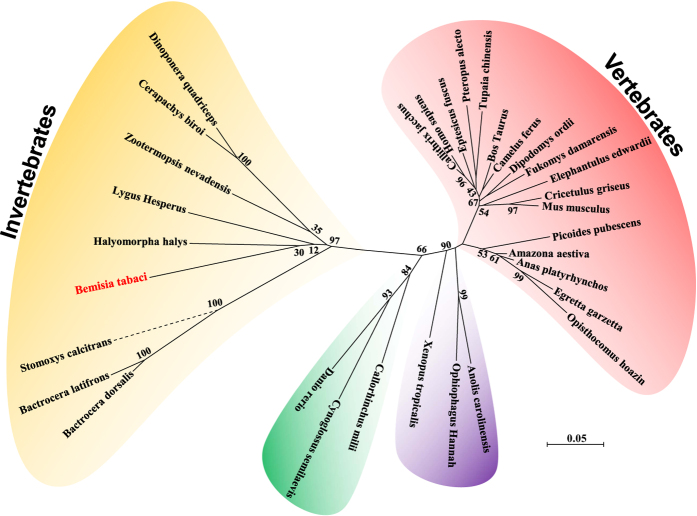
Phylogenetic tree of *HOT*. An unrooted phylogenetic tree showing the phylogenetic relationships of HOT genes from Insecta and non-Insecta species. The tree was generated by ClustalW alignment of the full-length amino acid sequences of HOT genes using the neighbor-joining (NJ) method in MEGA 6.0. Bootstrap values expressed as percentages of 1000 replications are shown at branch points.

The 3′ and 5′ RACE reactions were performed to determine complete the cDNA sequence of the *HOT* gene. Its cDNA contains a 1428-bp open reading frame encoding 475 amino acid residues. Genomic structure analysis exhibited that the *HOT* of *Bemisia tabaci* contains 11 exons (Fig. 1A). The predicted isoelectric point of the cDNA-deduced protein is 6.94, and the molecular weight (MW) is 51816. The deduced amino acid sequence contains important conserved domains that are common to other *HOT* members (Fig. 1B). The position Asp89 discriminates against NADP binding, while positions Asp250, His254, His335, and His365 are the metal-binding residues (Fig. 2). An unrooted phylogenetic tree showing the phylogenetic relationships of *HOT* genes from Insecta and non-Insecta species. The tree was generated by ClustalW alignment of the full-length amino acid sequences of *HOT* genes using the neighbor-joining (NJ) method in MEGA 6.0. Bootstrap values expressed as percentages of 1000 replications are shown at branch points. Homology analysis of amino acid sequence indicated that *HOT* shows highest pairwise amino acid similarity with *Halyomorpha halys HOT* (GenBank XP_014273839), i.e. 72.42% identity (Fig. 3).

### Relative expression levels in the developmental stages

**Figure 4:**
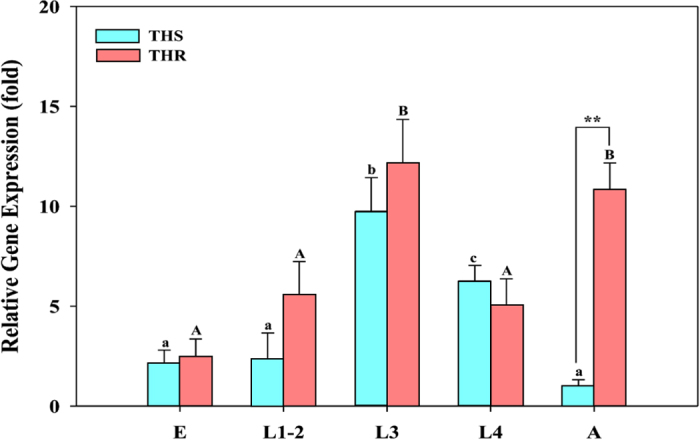
Relative expression levels of *HOT* in a thiamethoxam-sensitive strain (THS) and a thiamethoxam-resistant strain (THR) of *B. tabaci* as affected by developmental stage (E = eggs, L1-2 = first and second instar larvae, L3 = third instar larvae, L4 = fourth instar larvae, and A = adults). *RPL29* and *EF-1α* were used as internal reference genes to normalize data sets and calculate expression levels. The relative expression levels (fold-change) were calculated by assigning the lowest expression (hat to THS adults) a value of 1.0. Within each strain, different letters above the bars indicate significant differences in gene expression (P < 0.05; n = 3). **indicates a significant difference (P < 0.01) between the THS and THR strains.

The expression of *HOT* during different developmental stages of strains THR and THS of *B. tabaci* was examined by quantitative real-time polymerase chain reaction (qRT-PCR) (Fig. 4). In both THR and THS, *HOT* expression gradually increased from the egg to the second larval instar, dramatically increased in the third larval instar, and decreased in the fourth larval instar. *HOT* expression then declined to low levels in THS adults but increased to high levels in THR adults. *HOT* expression was significantly greater in THR adults than in THS adults.

### Expression in field populations

**Figure 5:**
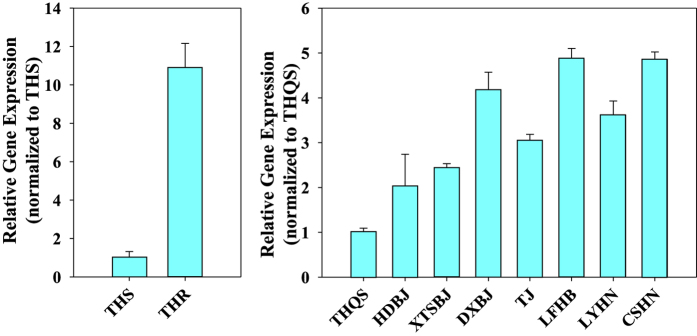
Expression profiles of HOT from *B. tabaci* representing 10 populations (populations codes listed along the X-axes) in China. Relative gene expression was measured by qRT-PCR. The Ct value for tested genes were normalized to the Ct value for *RPL29* and *EF-1α* and calculated relative to a calibrator using the formula 2^−ΔΔCt^. Values represent means ± SD for three independent replicates.

**Figure 6:**
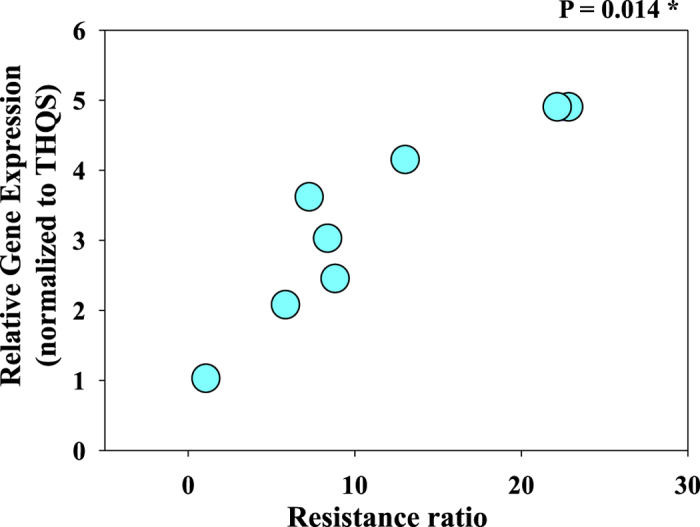
Linear regression analysis between resistance ratio and relative gene expression. Linear regression analysis was used to test for functional relationship between the resistance ratio of the strains and the mean normalized expression value of each gene in each strain. Significant regression lines (P ≤ 0.05) are marked with an asterisk.

qRT-PCR was used to compare the expression of *HOT* in seven field *B. tabaci* Q populations vs. in the *B. tabaci* Q susceptible reference population (THQS). Relative expression of *HOT* was high in DXBJ, LFHB, and CSHN (Table 1; Fig. 5). The correlation between relative expression values and resistance (RF values) was significant and positive for the gene of *HOT (P = *0.014, Fig. 6).

### Gene silencing for *Bemisia tabaci* adults

**Figure 7:**
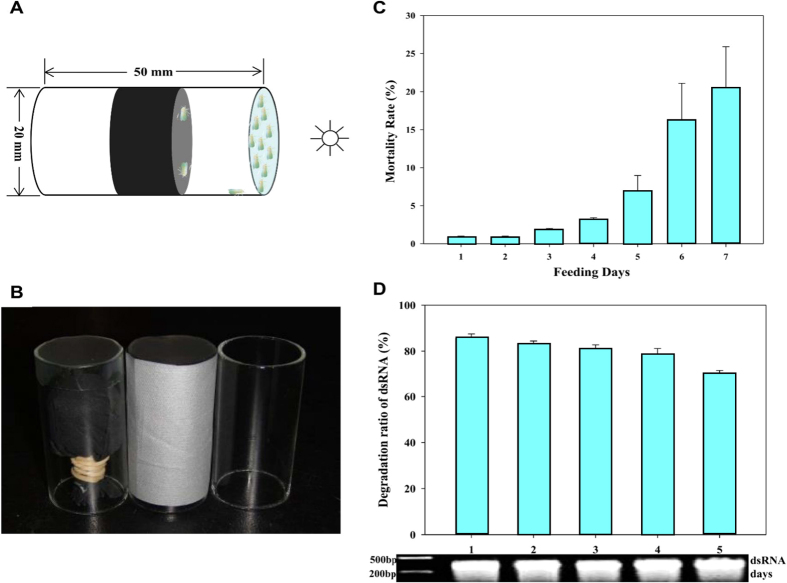
Gene silencing system for *B. tabaci* adults. (**A**,**B**) Gene silencing by supplying dsRNA in the diets of adults. The diet ± dsRNA is contained between two layers of Parafilm at one end of a glass feeding tube (20 mm in diameter × 50 mm long). After white flies are released into the other end of the tube, the tube is sealed with a black cotton plug, covered with a shade cloth, and oriented with the Parafilm-covered end toward a light source about 0.2 m away. (**C**) Mortality rate of *B. tabaci* after feeding on 30% sucrose without dsRNA for 7 days. (**D**) Stability of ds GFP in the sucrose solution contained between the two layers of Parafilm in the glass feeding chamber. The solution was sampled every day for 5 days, and the dsRNA was quantified by spectroscopy and examined by agarose gel electrophoresis.

A modified method used a feeding chamber in which dsRNA is supplied between two layers of Parafilm at the end of a glass feeding tube (Fig. 7A,B). To determine whether the chambers supported satisfactory whitefly survival, we assessed the mortality of *B. tabaci* adults that were supplied with sucrose lacking dsRNA for 7 days. The results showed that the mortality of these control adults increased slightly for the first 5 days and then increased rapidly on days 6 and 7 (Fig. 7C). We concluded that the system could be used for 5 days but not for longer periods. When the same system was used to evaluate the stability of *GFP* dsRNA contained in the membranes of the feeding chamber, the concentration of *GFP* dsRNA declined only slightly in 5 days (Fig. 7D). Hence, the system used to silence genes in adult whiteflies resulted in low levels of mortality and high dsRNA stability by day 5, and was subsequently used to knockdown genes in *B. tabaci.*

### RNA interference aided suppression of thiamethoxam resistance

**Figure 8:**
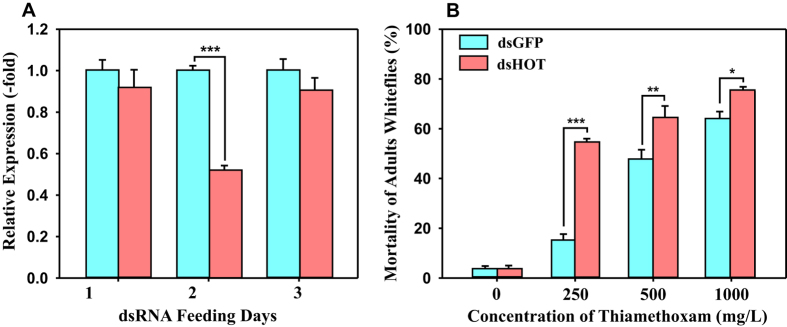
Effect of ds*HOT* (dsRNA of *HOT*) on expression of *HOT* in adults of a thiamethoxam-resistant strain (THR) and effect of *HOT* knockdown on resistance to thiamethoxam. (**A**) mRNA levels of *HOT* were quantified by qRT-PCR in 3 days of feeding on a diet containing dsGFP or ds*HOT*. The mRNA levels are shown as a ratio relative to the levels for the references genes *RPL29* and *EF-1α*. Values are means + SEMs (n = 3). As indicated by the asterisk, gene expression was significantly less (*P* < 0.05) with ds*HOT* than with dsGFP according to one-way ANOVAs. (**B**) Bioassays for THR adults exposed to thiamethoxam and for THR adults exposed to thiamethoxam in a diet with or without dsHOT or dsGFP. Mortality was assessed after 2 days of feeding. THR strain feeding to dsGFP as a control were exposed to 3 doses of thiamethoxam, and the mortality was recorded and graphed.

To further investigate the function of *HOT*, we used RNA interference (RNAi) technology to knockdown the expression of *HOT* in the THR strain. The results showed that the *HOT* mRNA levels decreased by 57% after 2 days of feeding on a diet containing *HOT* dsRNA (Fig. 8A), indicating that this gene was mostly silenced by RNAi. To determine whether knocking down the expression of *HOT* suppresses thiamethoxam resistance in the THR strain, we performed bioassays to compare thiamethoxam resistance levels among THR adults fed with *HOT* dsRNA or GFP dsRNA for 2 days. The results showed that thiamethoxam mortality remained low when adults were fed with GFP dsRNA but increased significantly when adults were fed with *HOT* dsRNA at three concentrations of this insecticide (250, 500 and 1000 mg/L) (Fig. 8B). Taken together, RNA interference-mediated knockdown of the *HOT* gene decreases thiamethoxam resistance in adults of the whitefly *Bemisia tabaci*.

## Discussion

High levels of resistance to thiamethoxam have been reported for *B. tabaci* in many countries. However, mechanisms of resistance to thiamethoxam in *B. tabaci* are poorly understood. Only a few genes, e.g., cytochrome P450s and GST and ABC transporters, have been associated with thiamethoxam resistance^16^,^17^. In the current study, which extends a previous study^13^, we cloned the full-length cDNA of *HOT* in *B. tabaci* and aligned the sequence with those of other *HOT* genes; the results demonstrated that the *B. tabaci HOT* is a classic *HOT* gene. The relative expression of *HOT* in different development stages was evaluated by qRT-PCR. The results showed that *HOT* expression was low in the egg and in the first and second larval instars but then increased substantially in the third larval instar. As development continued, *HOT* expression gradually decreased in the THS strain. In 2011, *B. tabaci* Q was detected in all seven locations sampled, and the expression of *HOT* was positively correlated with thiamethoxam resistance in field populations, compared with THQS population. the resuslt exhibited that the mechanism of thiamethoxam resistance mediated by *HOT* over-expression is very important in field populations.

A modified method to silence genes in whiteflies and other sap sucking insects would be very useful. Unlike direct injection of dsRNA^18^, the method described in the current study does not physically damage adult whiteflies. In addition, this method does not effect by plant endogenous, compared with a leaf-mediated dsRNA feeding method^19^. Because the mortality of the whiteflies in the feeding chambers dramatically increases on the sixth day, however, this method will not be suitable for the silencing of genes for long periods.

The current study documented a significant difference in *HOT* expression in *B. tabaci* adults of the THR vs. THS strain, and seven field populations. With whitefly development, *HOT* expression continued to drop to very low levels in THS adults but increased to high levels in THR adults, a finding which is in accord with the results obtained by Xie *et al*.^13^, who used the suppression subtractive hybridization cDNA library approach. When the modified RNAi system was used to knockdown the expression of *HOT* in the THR adults, the resistance level of thiamethoxam decreased significantly, indicating that over-expression of *HOT* plays an important role in thiamethoxam resistance.

The functions of *HOT* in insect is poorly understood, especially in resistance mechanisms. *HOT* catalyzes the cofactor-independent conversion of GHB to SSA, and that effect is coupled with the conversion of 2-oxoglutarate to D-2-hydroxyglutaric acid^14^. Previous research with *Apis mellifera* showed that γ-aminobutyric acid (GABA)-induced currents were partially blocked when imidacloprid was applied and that this block was independent of activation of nAChRs^20^. That result suggests that neonicotinoid insecticides may induce the inhibition of the GABA receptor in insects. The role of GABA as a major central nervous system (CNS) inhibitory neurotransmitter in the adult brain is well established^21^,^22^. In the current study, over-expression of *HOT* putatively increases the amount of GABA. A new hypotheses describe how high levels of *HOT* might contribute to thiamethoxam resistance in *B. tabaci*. According to the hypothesis, high levels of GABA induced by overexpression of *HOT* reverse the blocking of the GABA receptor by thiamethoxam, and long periods of exposure to thiamethoxam release the inhibition of GABA synaptic transmission^23^, resulting in desensitization of nAChRs and the stabilization of receptors in a closed state that is unresponsive to the insecticide^24^,^25^. Desensitized nAChRs are involved in several biological functions, such as neuroprotection, modulation of synaptic plasticity, and regulation of the release of neurotransmitters^26^. Several investigations have focused on the interactions between nAChR (α7) and GABA_A_ receptors. For example, at synaptic sites, the desensitization of presynaptic nAChR decreases the release of GABA for GABAergic neurons after repetitive exposure to nicotine^27^. α7-nAChR facilitates the TTX-sensitive release of GABA, which regulates the activities of pyramidal neurons^24^. A recent study indicated that brain GABA_A_ receptors is modulated negatively and positively by desensitized α7-nAChR as a result of choline pretreatment in cultured hippocampal neurons^28^.

In summary, a modified method of silencing genes in adult whiteflies was established in this paper. Using this method, we show that knockdown of the gene encoding *HOT* down-regulates *HOT* expression and substantially reduces thiamethoxam resistance in *B. tabaci* adults. We are conducting further studies on how over-expression of *HOT* affects GHB, and GABA, in order to elucidate the mechanism of *HOT*-induced resistance in *B. tabaci*. Although our findings indicate that thiamethoxam resistance involves *HOT*, our findings do not exclude the possibility that thiamethoxam resistance may also involve other mechanisms, such as the P450 monooxygenases and target-site resistance.

## Methods

### Insect populations

A thiamethoxam susceptible (THS, LC_50_ = 17.6 mg/L) and resistant (THR, LC_50_ = 1165 mg/L) strain of *B. tabaci* B were used in this study and reared as previously described (Table 1)^29^. The THS strain was collected from the greenhouse of the Institute of Vegetables and Flowers, Chinese Academy of Agricultural Science (CAAS) in 2000, and maintained in the greenhouse on cabbage (*Brassica oleracea L. var. capitata*) for more than 15 years without exposure to any insecticides. The THR strain was selected from the THS strain and exhibited an approximately 66-fold increase in thiamethoxam resistance relative to that of the THS strain^13^. A thiamethoxam-susceptible strain of *B. tabaci* Q (strain THQS, LC_50_ = 42 mg/L) was collected from Zhejiang province in 2011 and has been maintained in the glasshouse on pepper (zhosngjiao #4, *Capsicum annuuml*) for more than 3 years without exposure to insecticides. Field adults *B. tabaci* Q were collected from different locations in China in 2011. Approximately 1000 adults were collected per location, 400 of them were frozen in liquid nitrogen for 10 min, and stored at −80 °C. The rest of whiteflies were used in bioassays.

### Insecticide and bioassays

A bioassay was conducted with formulated thiamethoxam (250 g kg^−1^ WG; Syngenta China Investment Co. Ltd.). The bioassay was conducted according to the leaf dipping method described by Feng *et al*.^29^. The insecticide was dissolved and diluted with distilled water containing Triton X-100 (0.1‰). Leaf discs (22 mm diameter) from cabbage plants were dipped for 10 s in the insecticide solution or in distilled water (containing 0.1‰ triton X-100) as a control. After they were air dried, the leaf discs were placed with their adaxial surface downwards on agar (2 mL of 20 g/L) in a flat-bottomed glass tube (78 mm long). Adult whiteflies were collected by inverting the tubes above the leaves of the glasshouse cultures or tubes containing the artificial diet with dsRNA, so that adults (mixed sexes) would fly into the tube. The open end of the tube was then sealed with a cotton plug. Each tube contained 15–30 adults and was kept in an incubator at 25 °C with a 14:10 (L:D) p*HOT*operiod. Mortality was recorded after 48 h, and each combination of strain and thiamethoxam concentration was represented by four replicate tubes.

### RNA extraction, cDNA and dsRNA synthesis

Total RNAs from THR and THS were extracted from 40 *B. tabaci* adults (mixed sex) using a Trizol reagent (Invitrogen, Carlsbad, CA, USA) and following the manufacturer’s protocol. The resulting total RNA was resuspended in nuclease-free water and quantified with a spectrophotometer (Thermo Scientific Nanodrop 2000). Then, 1.0 μg of RNA of each sample was used to synthesize the first-strand cDNA using the PrimeScript^®^RT reagent Kit with gDNA Eraser (Perfect Real Time) (Takara Bio, Tokyo, Japan) according to the manufacturer’s protocol. Synthesis of *HOT* and GFP dsRNA and application of RNAi to insect were carried out according to published protocols^30^. In addition, dsRNAs were prepared using the T7 RiboMAX Express RNAi system and protocols (Promega, WI, USA). Purified dsRNAs were quantified by spectroscopy and examined by agarose gel electrophoresis to ensure their integrity. The dsRNA was stored at −80 °C before use.

### Quantitative real-time PCR

A total of 120 *B. tabaci* adults (three biological replicates, n = 40) from THR and THS were subjected to qRT-PCR analysis. Primers were designed to amplify a 107-bp fragment as listed in Table 2. The 25-μl reaction system consisted of 1 μl of diluted cDNA, 11.25 μl of SYBR^®^ Green Real-time PCR Master Mix (TIANGEN, Corp, Beijing, China), and 0.5 μl of each primer. Real-time PCR was conducted with the ABI 7500 system using the following protocol: 3 min of activation at 95 °C followed by 40 cycles of 40 s at 95 °C, 30 s at 60 °C, and 32 s at 72 °C. A 3-fold dilution series of cDNA was used to construct a relative standard curve and PCR efficiency, which was used to convert threshold cycle (Ct-values) into raw data (relative quantities). The qRT-PCR primer efficiency of *HOT* is 96.4%. The fold-changes in *HOT* gene expression, normalized to the reference genes *EF-1α* and *RPL29*^31^, were calculated using the 2^−ΔΔCt^ method^32^.

### Rapid amplification of cDNA ends (RACE)

Xie *et al*.^13^. reported that an EST sequence of *HOT* is over-expressed in the THR strain of *B. tabaci*. The 3′ and 5′ RACE reactions were constructed using the SMART^TM^ RACE amplification kit (Clontech, USA) according to the manufacturer’s protocol. All primers used are listed in Table 2. The PCR products were cloned into the pMD18-T simple vector (TaKaRa, Japan). At least 10 clones from both the 5′- and 3′-RACE libraries were sequenced using both M13 forward and M13 reverse primers.

### Gene silencing system

Gene silencing was achieved by directly feeding dsRNA to whitefly adults in a feeding chamber^33^. The feeding chamber consisted of a glass tube (20 mm in diameter × 50 mm long, open at both ends), which was covered at the top end by one layer of Parafilm-membrane (Alcan Packaging, Chicago, IL, USA) stretched as thinly as possible. A 0.25-mL drop of diet solution (5% yeast extract and 30% sucrose (wt/vol)) was placed on the outer surface of the stretched Parafilm and was covered with another layer of stretched Parafilm to enclose the solution between the Parafilm layers. Whiteflies were released into the other end of the tube. Then the tube was sealed with a black cotton plug and covered with a shade cloth. The end of the tube with the Parafilm membranes was turned toward a light source that was approximately 0.2 m away (Fig. 7A,B). In an initial experiment, we determined whether the feeding chambers supported the survival of adult *B. tabaci*. Feeding chambers were prepared as described in the previous paragraph but without dsRNA, i.e., the adults were fed only the sucrose solution. Whitefly mortality was assessed every day for 7 days. In another initial experiment, we determined the rate at which dsRNA degraded in the feeding chambers. dsGFP (0.5 μg/μL) was placed in each of 15 replicate feeding chambers (without whiteflies), and the quantity of dsGFP remaining in each of three replicate chambers was quantified every day for 5 days with a spectrophotometer (Thermo Scientific Nanodrop 2000).

This modified system was used to measure how feeding on dsHOT affected the expression of *HOT* and thiamethoxam resistance in adult *B. tabaci.* The control diet solution was 5% yeast extract, 30% sucrose (wt/vol) and dsGFP (0.5 μg/μL), and the treatment diet was the same basic solution with the addition of dsHOT (0.5 μg/μL). Approximately 40 newly emerged adults (mixed sexes) were introduced into each feeding chamber, which were placed in an environmental chamber (Panasonic MLR-352H, Gunma, Japan) at 25 °C, a photoperiod of L14 : D10, and 80% RH^34^,^35^.

### Data analysis

For determination of bioassay data were analysed by probit analysis using the POLO program PC PoloPlus (Leora Software, Berkeley, CA). Mortality was corrected using Abbott’s formula for each probit analysis^36^. Results of the qRT-PCR analysis are presented as means and standard errors. Statistical significance was evaluated using one-way ANOVAs at the 0.05 level followed by the LSD test. Linear Model II regression analysis was used to test for functional relationship between the resistance level of the strains and the mean normalized expression value of each gene in each strain by using SPSS^24^ (SPSS for Windows, Rel. 17.0.0 2009. Chicago: SPSS Inc.).

## Additional Information

**How to cite this article:** Yang, X. *et al*. RNA interference-mediated knockdown of the hydroxyacid-oxoacid transhydrogenase gene decreases thiamethoxam resistance in adults of the whitefly *Bemisia tabaci. Sci. Rep.* **7**, 41201; doi: 10.1038/srep41201 (2017).

**Publisher's note:** Springer Nature remains neutral with regard to jurisdictional claims in published maps and institutional affiliations.

## Figures and Tables

**Table 1:** Responses of field populations of *Bemisia tabaci* from China to thiamethoxam in the laboratory bioassay.

Population	N^a^	Slope ( ± SE)	df^b^	LC_50_ (mg L^−1^) (95%FL^c^)	RR^d^
THS	678	2.16( ± 0.021)	4	19.12(14.52~25.13)	—
THR	745	2.90( ± 0.16)	4	1426.27(1259.12~1615.49)	74.6
THQS	358	1.16( ± 0.15)	4	42.13(20.89~89.12)	—
Haidian Beijing (HDBJ)	252	2.53( ± 0.15)	3	251.16(214.58~293.69)	5.96
Xiaotangshan Beijing (XTSBJ)	304	2.50( ± 0.17)	4	370.14(296.65~461.84)	8.78
Daxing Beijing (DXBJ)	341	2.28( ± 0.22)	4	554.87(417.4~737.61)	13.17
Tianjing (TJ)	426	1.81( ± 0.13)	4	353.54(288.72~432.91)	8.39
Langfang Hebei (LFHB)	294	1.63( ± 0.24)	4	946.81(579.45~1547.05)	22.47
Luoyang Henan (LYHN)	324	1.33( ± 0.19)	3	305.58(172.13~542.49)	7.25
Changsha Hunan (CSHN)	461	1.32( ± 0.17)	5	938.46(508.06~1733.47)	22.28

^a^N = numbers of *B. tabaci* in each bioassay.

^b^df = Degree of freedom.

^c^FL = fiducial limit.

^d^RR (resistance Ratio) = LC_50_ of the sample population)/LC_50_ of population WHHB.

**Table 2:** Primers used in this study.

Putative gene	Primers 5′-3′*	Annealing temperature (°C)	Product (bp)
3′GSP	ATGCTGTGAAGGACTCGAGGGCATG	70.5	—
3′NGSP1	AGGCAGTGAAACGACGGGTGTTAGT	67.3	—
3′NGSP2	GTCCTTCACAGCATTGCCCTACACA	67.3	—
5′GSP	CATGCCCTCGAGTCCTTCACAGCAT	70.5	—
5′NGSP1	CTGTGTAGGGCAATGCTGTGAAGGA	67.4	—
5′NGSP2	TCGGAGATGGGATTGCTTCCTTGAT	69.1	—
*HOT*-full length (GenBank KF439724)	F-TACCATTGCGGCTACAACATACTR-GCCTCGGGGCAAGTTTAGTGATT	59	1563
*HOT*-qPCR	F-TCGAGTCCTTCACAGCATTGCR-CCATCTCCGATATTTGGGCC	60	107
*EF-1α*(GenBank EE600682)	F-TAGCCTTGTGCCAATTTCCGR-CCTTCAGCATTACCGTCC	60	110
*RPL29*(GenBank EE596314)	F-TCGGAAAATTACCGTGAGR-GAACTTGTGATCTACTCCTCTCGTG	60	144

^*^F, forward primer; R, reverse primer.
